# Road Profile Estimation Using a 3D Sensor and Intelligent Vehicle

**DOI:** 10.3390/s20133676

**Published:** 2020-06-30

**Authors:** Tao Ni, Wenhang Li, Dingxuan Zhao, Zhifei Kong

**Affiliations:** 1 School of Mechanical and Aerospace Engineering, Jilin University, Changchun 130022, China; nitao@jlu.edu.cn (T.N.); whli19@mails.jlu.edu.cn (W.L.); kongzf17@mails.jlu.edu.cn (Z.K.); 2 School of Mechanical Engineering, Yanshan University, Qinhuangdao 066004, China

**Keywords:** autonomous vehicle, laser measurement, model predictive control, measurement uncertainty

## Abstract

Autonomous vehicles can achieve accurate localization and real-time road information perception using sensors such as global navigation satellite systems (GNSSs), light detection and ranging (LiDAR), and inertial measurement units (IMUs). With road information, vehicles can navigate autonomously to a given position without traffic accidents. However, most of the research on autonomous vehicles has paid little attention to road profile information, which is a significant reference for vehicles driving on uneven terrain. Most vehicles experience violent vibrations when driving on uneven terrain, which reduce the accuracy and stability of data obtained by LiDAR and IMUs. Vehicles with an active suspension system, on the other hand, can maintain stability on uneven roads, which further guarantees sensor accuracy. In this paper, we propose a novel method for road profile estimation using LiDAR and vehicles with an active suspension system. In the former, 3D laser scanners, IMU, and GPS were used to obtain accurate pose information and real-time cloud data points, which were added to an elevation map. In the latter, the elevation map was further processed by a Kalman filter algorithm to fuse multiple cloud data points at the same cell of the map. The model predictive control (MPC) method is proposed to control the active suspension system to maintain vehicle stability, thus further reducing drifts of LiDAR and IMU data. The proposed method was carried out in outdoor environments, and the experiment results demonstrated its accuracy and effectiveness.

## 1. Introduction

In the last three decades, driverless technology has rapidly developed due to people’s willingness to improve living standards, and the need to improve work efficiency. The key issue to driverless vehicles is how to drive to a given place without any traffic accidents. The localization and perception of autonomous vehicles are two significant technological aspects to solve the above issue. Most studies on driverless vehicles used multi-sensors such as inertial measurement units (IMUs), light detection and ranging (LiDAR), and global navigation satellite systems (GNSSs) to achieve vehicle perception and localization [1,2,3,4,5]. Gao [6] proposed a robust localization system that made better use of the relative merits of LiDAR and global positioning systems (GPSs) to correct the data obtained by inertial navigation system (INSs) selectively in outdoor and indoor environments. Experiment results showed that the proposed localization system could maintain meter-level localization accuracy within the experimental period. Wan [7] proposed a robust and precise localization method that could maintain centimeter-level navigation accuracy in some challenging environments. The proposed system used a Kalman filter (KF) algorithm to fuse the data obtained by physical sensors such as GPS, INS, and LiDAR to achieve high localization accuracy and robustness. Wolcott [8] proposed a robust localization system of driverless vehicles based on gaussian probabilistic model. Results showed that the proposed system significantly improved the localization accuracy compared to other methods. Tang et al. [9] presented a navigation system that takes advantage of the complementary characteristics of INS and LiDAR. The proposed system frame used an extended Kalman filter (EKF) algorithm to fuse the information from LiDAR and INS, which ensured that the advantages of each subsystem were fully utilized to achieve long-term stable localization accuracy of autonomous vehicles. Chang [10] proposed a simultaneous localization and mapping (SLAM) navigation system that fused the information obtained by GNSS, INS, and LiDAR to achieve vehicle mapping and localization. The advantage of the proposed method is that it estimated pose information based on graph optimization technology. Experiment results showed that the method could effectively utilize data from GNSS, INS, and LiDAR, and improve the localization accuracy even when the INS data and GNSS signal are unstable. Qin [11] presented a visual-inertial navigation system that used nonlinear analysis and graph optimization technology to obtain accurate and robust pose estimation. Qian [12] investigated a SLAM algorithm that effectively used heading angle and velocity information from INS/GPS to improve the navigation accuracy of vehicles. Experimental results showed that the proposed method achieved centimeter-level horizontal localization accuracy. The authors in [13] proposed a simultaneous localization and mapping method of constructing forest maps using LiDAR, IMU, and GPS. The proposed method generated a 3D map using cloud data points obtained by 3D laser scanners and improved it with a graph optimization algorithm. 

Accurate road profile estimation is significant for autonomous vehicles driving on uneven terrain, as it can be used as a reference index for vehicle path planning. Several methods on road profile estimation have been proposed. The authors in [14] used 3D laser scanners to obtain road and environment information for mapping. The presented approach processed cloud data points with a segmentation algorithm and calculated pose information from the obtained local maps. Droeschel [15] presented a simultaneous localization and mapping system that built local dense 3D maps of surrounding environments with a graph-optimization algorithm. Zhao [16] proposed a method for obtaining accurate road profile information using 3D laser scanners, INS, and GPS. The proposed method fused the information from GPS and INS using a KF algorithm to obtain accurate and robust pose information. Peter [17] proposed a novel method for terrain profile estimation that fully considered the errors of measurement system and incorporated uncertainties of 3D laser scanner and INS measurements for yielding a probabilistic elevation map. Wang [18] proposed a method for constructing the terrain profile and extracting preview elevation values based on predicted pose information of vehicles. Results demonstrated that the proposed algorithm achieved centimeter-level accuracy for the estimated elevation map.

The stability of vehicles is an important prerequisite for obtaining high-precision terrain information. However, violent vibrations are inevitable, even with appropriate path planning, for autonomous vehicles driving on uneven roads. One way to solve the problem is to equip the vehicle with an active suspension system to maintain stability when driving on complex terrain. The active suspension system is a significant component of vehicles because it can generate the force between the vehicle body and wheel to compensate vehicle vibration caused by terrain unevenness. A wide range of suspension controllers were proposed [19,20,21,22]. Huang [23] presented an adaptive controller for vehicle active suspension systems to solve the problem of parameter uncertainties. Marzbanrad [24] proposed an optimal control method for vehicle suspension system on uneven terrain. Road elevation values were calculated in advance and as input information to control active suspension systems in this paper. Pan [25] investigated an adaptive control method for active suspension systems to improve ride comfort and handling stability. Youn et al. [26] investigated a preview control method for the semi-active and active suspension systems. The road disturbance information was obtained from the front wheels and used as a preview input of rear wheels of the vehicle. Göhrle [27] proposed a novel preview control method that obtained terrain profile information using a vehicle equipped with ranging sensors and controlled active suspension systems using this information to improve vehicle performance. Result showed that the terrain profile information could be well estimated, and ride comfort and handling stability were also improved with the proposed method. Theunissen et al. [28] proposed an explicit model predictive control method that used the road information obtained by LiDAR as the signal input to control the active suspension system. Experiment results showed that the heave and pitch acceleration of the sprung mass was reduced compared with the passive suspension system and skyhook controllers. The proposed algorithm also required less memory space compared with other methods, because the complex calculation process was operated offline. Du [29] proposed an output feedback control method for vehicle suspension systems that used a nonlinear extended state observer to estimated model uncertainties. The proposed approach could effectively estimate the nonlinear dynamics and mismatch disturbances such as sprung mass, unknown friction coefficient, and measurement noise caused by sensors. Results showed that vehicle ride comfort was significantly improved, and the proposed algorithm could achieve fast convergence, which indicated that the method was more applicable in engineering practice. Sun [30] investigated an active suspension controller that considered the impact of actuator input delay and band with constraints. Experiment results showed that the proposed approach achieved better vehicle performance compared with the traditional approach for active suspension systems. Pan [31] proposed an output feedback finite-time controller for vehicle active suspension systems. The proposed method used a robust first-order sliding mode observer to compensate for the model uncertainties such as mismatch disturbances, unknown parameter, and unmodeled dynamics. Results showed that the proposed controllers could effectively decrease root-mean-square errors of the vehicle vertical velocity and acceleration.

Although the aforementioned methods on road profile estimation could achieve good effects in some scenes, they could cause large errors for a vehicle working on complex uneven terrain. There are also a few scholars who conducted detailed studies on autonomous vehicles driving on complex terrain. For the above motivation, we propose a method for road profile estimation and active suspension control on complex uneven terrain. In Section 2, we introduce the configuration of our autonomous vehicle and the overall flow of our approach. In Section 3, details about the terrain profile estimation method are shown. In Section 4, we propose a method for model predictive control (MPC) with a preview, which uses terrain profile information and IMU/GPS data to control the active suspension system. Lastly, real-world experiments were carried out to demonstrate the accuracy and effectiveness of our approach.

## 2. System Structure

**Figure 1:**
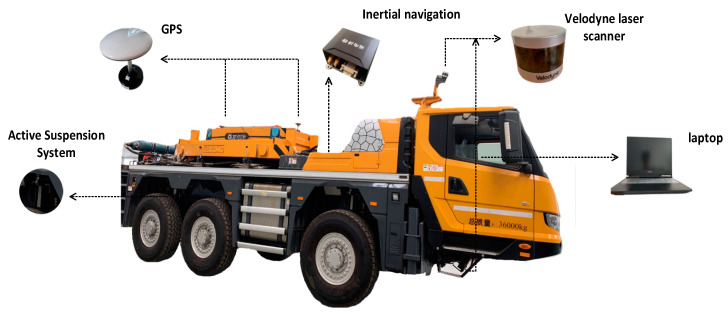
Vehicle configuration.

**Figure 2:**
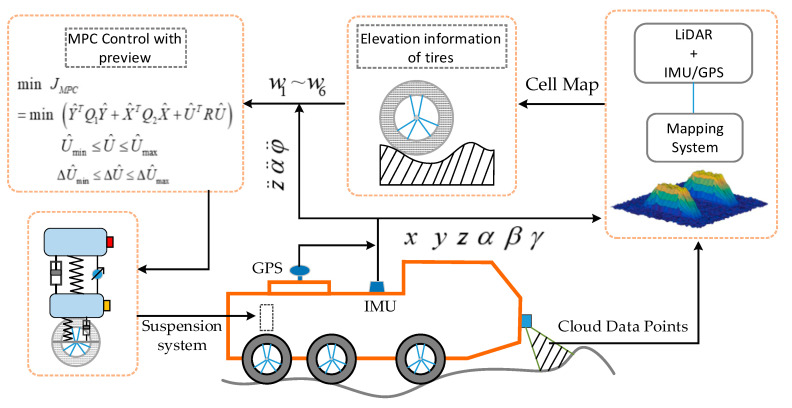
System structure.

Autonomous vehicles working on uneven terrain need to consider not only the surrounding environment but also terrain profile information, which is different from autonomous vehicles driving on urban even driveways. Accurate road profile information can be used as a reference index for vehicle-path planning. However, the traditional autonomous vehicles experience violent vibrations when driving on uneven terrain, which leads to drifts of LiDAR and IMU data. Therefore, how to obtain accurate terrain information and drive autonomous vehicles stably and safely on complex roads are two significant issues. In order to solve the above issue, our vehicle was equipped with two GPSs, an IMU, two 3D laser scanners, and an active suspension system, as shown in Figure 1. Location accuracy is a prerequisite for constructing an accurate road profile map. Therefore, our system used real time kinematic (RTK) technology to ensure centimeter-level positioning accuracy of GPS. Our vehicle also serves for open outdoor scenes, so the stability of the positioning system is well-assured. With accurate positioning information, cloud data points obtained by LiDAR were added to the terrain elevation map and further processed by the Kalman filter algorithm. Ultimately, the elevation map information and IMU/GPS data were used as inputs to control the active suspension system to maintain vehicle stability; Figure 2 shows the main steps. 

## 3. Method

### 3.1. Elevation Map Estimation 

Constructing a reliable and accurate elevation map needs to consider the systematic error of sensor measurements. In this section, we propose a method for constructing a probability elevation map. Elevation information in each cell of the map includes not only the estimated evaluation value, but also the confidence boundary of the estimated value. Our method is different than other proposed methods (proposed in [17]) for probability map estimation because our autonomous vehicle has an active suspension system and a global positioning system that can ensure the stability and positioning accuracy of the vehicle, thus further reducing sensor drifts.

#### 3.1.1. Coordinate System Definition

**Figure 3:**
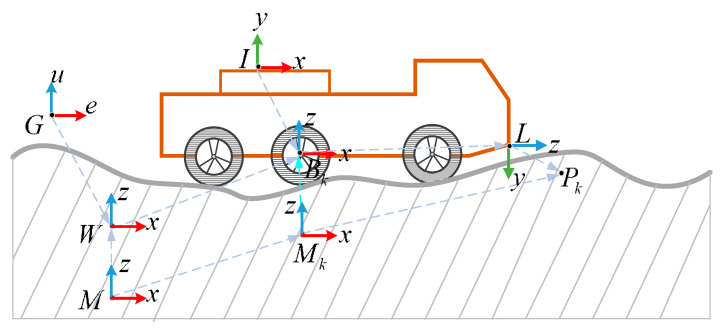
Automobile coordinate system.

Seven coordinate systems were defined in our autonomous vehicle, namely East-North-Up (ENU) coordinate system G, inertial coordinate system W, map coordinate system M, vehicle coordinate system $B_{k}$, IMU coordinate system I, local map coordinate system $M_{k}$, and LiDAR coordinate system L, respectively, as shown in Figure 3.

Suppose that the inertial coordinate system W is fixed in the real environment. Vehicle coordinate system $B_{k}$, IMU coordinate system I, and LiDAR coordinate system L are fixed in the corresponding position of the vehicle, respectively, and their position relationship can be obtained through calibration technology. Map coordinate system M is also fixed in the real environment, as is the inertial coordinate system W. Local map coordinate system $M_{k}$ moves with the motion of the vehicle and maintains an invariant transformation with vehicle coordinate system $B_{k}$. 

#### 3.1.2. Uncertain Estimation of Location System 

**Figure 4:**
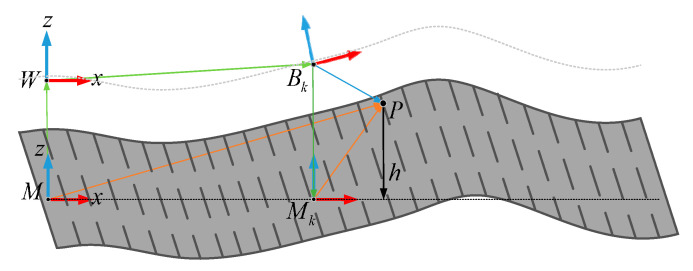
Automobile sub-coordinate system.

The motion of the vehicle can create noise errors because of systematic errors caused by GPS/IMU, which influence the precision of the elevation map. Therefore, errors caused by GPS/IMU need to be considered when constructing the elevation map. A sub-coordinate system was built to describe the coordinate transformation relationship, as shown in Figure 4. Measurement value P expressed in coordinate system $B_{k}$ at time k can be converted to map coordinate system M:

(1) RMP,kM=RMWM+RWP,kM=RMWM+RWP,kW=RMWM+RWBkW+ΦBkW−1(RBkPBk)

where ΦBkW−1(RBkPBk) represents the rotation transformation of vector $R_{B_{k}P}$ from coordinate system $B_{k}$ to coordinate system W.

The Gaussian probability distribution of $R_{WB_{k}}$ and $\Phi_{WB_{k}}$ can be defined as follows:

(2) $$\left\{ \begin{array}{l} R_{WB_{k}}∼N({\hat{R}}_{WB_{k}},\sum {}_{r,k})\\ \Phi_{WB_{k}}∼N({\hat{\Phi }}_{WB_{k}},\sum {}_{\Phi ,k}) \end{array}\right.$$

where $\sum {}_{r,k}$ and $\sum {}_{\Phi ,k}$ represent the covariance of the pose estimation between the inertial coordinate system W and the vehicle coordinate system $B_{k}$.

Then, the error propagation of the location system can be calculated as follows [17]:

(3) $$\sum {}_{move,k}=J_{r,k}\sum {}_{r,k}J_{r,k}^{T}+J_{\Phi ,k}\sum {}_{\Phi ,k}J_{\Phi ,k}^{T}$$

With Equation (1), the Jacobian matrices can be obtained:

(4) {Jr,k=∂MRMP,k∂RWBkW=∂RWBkW∂RWBkW+∂(RMWW+ΦBkW−1(RBkPBk))∂RWBkW=IJΦ,k=∂RMP,kM∂ΦBkW=∂(RWBkW+RMWW)∂ΦBkW+∂ΦBkW−1(RBkPBk)∂ΦBkW=C(ΦBkW)T(RBkPBk)×

#### 3.1.3. Uncertain Estimation of LiDAR System

From Figure 3, measurement point $P_{k}$ is generally represented by vector RLPkL in LiDAR coordinate system L. With the transformation from LiDAR coordinate system L to vehicle coordinate system $B_{k}$, the vector RBkPkBk could be obtained:

(5) RBkPkBk=ΦLBk−1(RLPkL)+RBkLkBk=ΦLB−1(RLPkL)+RBLB

The error propagation of the measurement system can be calculated as follows [17]:

(6) $$\sum {}_{L,k}=J_{L,k}\sum {}_{M,k}J_{L,k}^{T}$$

where $\sum {}_{M,k}$ represents the covariance matrix of the LiDAR measurement model, which can be obtained through sensor calibration technology.

The Jacobian matrix can be obtained according to Equation (5):

(7) JL,k=∂RBkPkBk∂RLPkL=∂ΦLB−1(RLPkL)∂RLPkL+∂RBLB∂RLPkL=C(ΦLB)T

The truncation error needs to be considered when the cloud data points are added to the corresponding grid cell of the elevation map. In order to reduce computation, the truncation error is set as an invariant value:

(8) σx2=σy2=(d2)2

Therefore, covariance matrix $\sum {}_{P}$ of each grid cell can be defined as follows:

(9) $$\sum {}_{P}=\left[\begin{array}{ccc} \sigma_{x}^{2} & 0 & 0\\ 0 & \sigma_{y}^{2} & 0\\ 0 & 0 & 0 \end{array}\right]$$

In summary, according to Equations (3), (6), and (9), the error propagation model of an autonomous vehicle system can be expressed as follows:

(10) $$\sum {}_{k}=\sum {}_{move,k}+\sum {}_{L,k}+\sum {}_{P}$$

#### 3.1.4. Elevation Map Update Method 

Cloud data points obtained by LiDAR at different times may be added to the same cell of the elevation map with the motion of the vehicle, so an effective algorithm is needed to fuse the cloud data points of the same cell. In this study, the Kalman filter algorithm was used to fuse the cloud data points, which not only improved measurement accuracy, but also effectively utilized the height value variance of each measurement point.

Suppose that the height measurement value and corresponding covariance matrix of a cell are $\hat{h}$ and $\sigma_{\hat{h}}^{2}$, respectively, and the height estimation value and corresponding variance matrix are $h_{k}$ and $\sigma_{h,k}^{2}$, respectively. The update process of the Kalman filter can be calculated:

(11) $$\left\{ \begin{array}{l} K_{k+1}=\sigma_{h,k}^{2}{\left(\sigma_{h,k}^{2}+\sigma_{\hat{h}}^{2}\right)}^{-1}\\ h_{k+1}=h_{k}+K_{k+1}(\hat{h}-h_{k})\\ \sigma_{h,k+1}^{2}=(I-K_{k+1})\sigma_{h,k}^{2} \end{array}\right.$$

With Equation (11), the following equation can be obtained:

(12) $$\left\{ \begin{array}{l} h_{k+1}=\frac{\sigma_{h,k}^{2}\hat{h}+\sigma_{\hat{h}}^{2}h_{k}}{\sigma_{h,k}^{2}+\sigma_{\hat{h}}^{2}}\\ \sigma_{h,k+1}^{2}=\frac{\sigma_{h,k}^{2}\sigma_{\hat{h}}^{2}}{\sigma_{h,k}^{2}+\sigma_{\hat{h}}^{2}} \end{array}\right.$$

Sometimes, in the elevation value in the same cell, large drops occur due to vertical objects in the surrounding environment. If the elevation value of the cell is fused by a Kalman filter without any judgment, the updated elevation value is far from the real value. In order to avoid the above case, this study used Mahalanobis distance to judge all measured values before elevation information fusion. Only when the Mahalanobis distance between the estimated elevation value and the new measured value is less than threshold value c is the new measured value added to the cell. The Mahalanobis distance criterion is defined as follows:

(13) $$d_{m}(Z_{k},Z_{k+1})=\sqrt{\frac{{\left(\hat{h}-h_{k+1}\right)}^{2}}{\sigma_{h,k+1}^{2}+\sigma_{\hat{h}}^{2}}}$$

(14) $$\left\{ \begin{array}{l} Z_{k}∼N(\hat{h},\sigma_{\hat{h}}^{2})\\ Z_{k+1}∼N(h_{k+1},\sigma_{h,k+1}^{2}) \end{array}\right.$$

(1) If $\hat{h}>h_{k+1}$ and $d_{m}(Z_{k},Z_{k+1})>c$, then:

(15) $$\left\{ \begin{array}{l} {\hat{h}}_{k+1}=\hat{h}\\ {\hat{\sigma }}_{h,k+1}^{2}=\sigma_{\hat{h}}^{2} \end{array}\right.$$

(2) If $\hat{h}<h_{k+1}$ and $d_{m}(Z_{k},Z_{k+1})>c$, then:

(16) $$\left\{ \begin{array}{l} {\hat{h}}_{k+1}=h_{k+1}\\ {\hat{\sigma }}_{h,k+1}^{2}=\sigma_{h,k+1}^{2} \end{array}\right.$$

(3) If $d_{m}(Z_{k},Z_{k+1})<c$, then the new measured value can be calculated with Equation (12).

**Figure 5:**
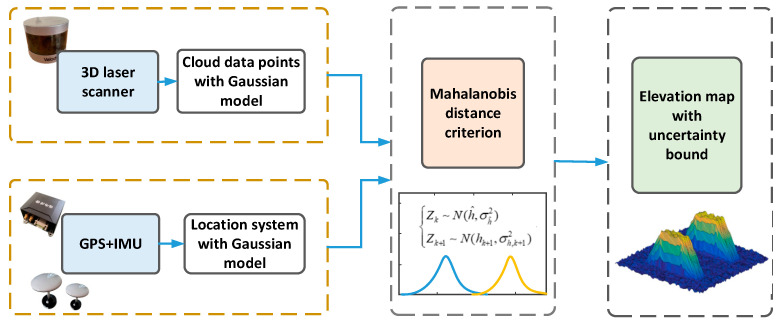
Probability map estimation system.

In summary, this section proposed a method for elevation map estimation that considers systematic errors caused by GPS/IMU, LiDAR, and map cells. The error-propagation model of vehicle systems was defined to calculate the confidence boundary of the estimated elevation value. Besides, the Kalman filter algorithm and Mahalanobis distance criterion were used to fuse the cloud data points of the same cell. The main flow of elevation map estimation is shown in Figure 5. 

### 3.2. Model Predictive Control with Preview

**Figure 6:**
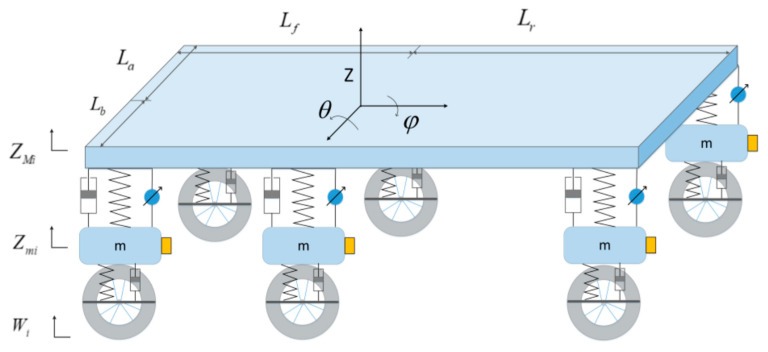
Autonomous vehicle model.

Our autonomous vehicle was equipped with an active suspension system for solving the problem of vehicle stability when driving on complex uneven terrain, which could be sampled as a nine-degree-of-freedom vehicle model, as shown in Figure 6. The vehicle model consisted of six vertical unsprung masses and degrees of freedom due to the pitch, roll, and vertical motion of the mass center.

According to Newton’s second law, the vertical motion of the body centroid, the pitching rotation, and the roll rotation equations can be obtained as:

(17) $$\left\{ \begin{array}{l} M\ddot{Z}=-F_{1}-F_{2}-F_{3}-F_{4}-F_{5}-F_{6}\\ J_{YY}\ddot{\theta }=L_{r}(F_{1}+F_{2})-L_{f}(F_{5}+F_{6})\\ J_{XX}\ddot{\varphi }=L_{a}(F_{1}+F_{3}+F_{5})+L_{b}(-F_{2}-F_{4}-F_{6}) \end{array}\right.$$

where $F_{i}$ is the force generated by the actuator; Z, the vertical displacement of vehicle centroid; θ, the pitch angle of vehicle centroid; φ, the roll angle of vehicle centroid; and $J_{XX}$ and $J_{YY}$, the moments of inertia of the X and Y axes, respectively. $L_{r}$ and $L_{f}$ are the distance between the center of mass, and front and rear suspension, respectively. $L_{a}$ and $L_{b}$ are the distance between center of mass, and left and right suspension, respectively.

In the preceding equations, $F_{i}$(i= 1, 2, 3, 4, 5, 6) acting as the force between the suspension and the vertical body can be calculated as follows:

(18) $$F_{i}=K_{i}(Z_{Mi}-Z_{mi})+C_{i}({\dot{Z}}_{Mi}-{\dot{Z}}_{mi})-f_{i}(t)$$

where $K_{i}$ is stiffness coefficients, $C_{i}$ is damping coefficients, and $f_{i}(t)$ is the force generated by actuators. In order to improve the flexibility of vehicle-suspension control, our vehicle-suspension system only consisted of actuators, as shown in Figure 1. Thus, stiffness coefficient $K_{i}$ and damping coefficient $C_{i}$ were set to zero.

The dynamic equation of the vertical motion of the unsprung mass is as follows:

(19) $$\begin{array}{cc} m_{i}{\ddot{Z}}_{mi} & =K_{i}(Z_{Mi}-Z_{mi})+C_{i}({\dot{Z}}_{Mi}-{\dot{Z}}_{mi})-f_{i}(t)\\ & \;+K_{mi}(Z_{mi}-W_{i})+C_{mi}({\dot{Z}}_{mi}-{\dot{W}}_{i}) \end{array}$$

where $K_{mi}$ and $C_{mi}$ are the stiffness and damping coefficients of the tires, respectively.

According to the spatial motion law of rigid bodies, the dynamic relationship among the six suspension systems and the body centroid can be obtained as:

(20) $$\left\{ \begin{array}{l} Z_{M1}=Z+L_{r}\sin\theta +L_{a}\sin\varphi \\ Z_{M2}=Z+L_{r}\sin\theta -L_{b}\sin\varphi \\ Z_{M3}=Z+L_{a}\sin\varphi \\ Z_{M4}=Z-L_{b}\sin\varphi \\ Z_{M5}=Z-L_{f}\sin\theta +L_{a}\sin\varphi \\ Z_{M6}=Z-L_{f}\sin\theta -L_{b}\sin\varphi \end{array}\right.$$

Suppose that the pitch and roll angles are sufficiently small, then the Equation (20) can be rewritten as follows:

(21) sinθ≈θ,sinφ≈φ

The Equations (17)–(21) can be rewritten into a state-space formulation:

(22) $$\left\{ \begin{array}{l} {\dot{X}}_{M}(t)=A_{M}X_{M}(t)+B_{M}U(t)+E_{W}W(t)\\ Y_{M}(t)=C_{M}X_{M}(t)+D_{M}U(t) \end{array}\right.$$

where $X_{M}(t)$ and $Y_{M}(t)$ denote state and output vectors, respectively; $A_{M}$, $B_{M}$, $C_{M}$, and $D_{M}$ are system matrices; $E_{W}$ is the road disturbance matrix; and W(t) is the road disturbance, which can be obtained by the estimated elevation map.

State and output vectors can be expressed as follows:

(23) $$\left\{ \begin{array}{l} X_{M}={\left[Z,\theta ,\varphi ,Z_{M1}\sim Z_{M6},\dot{Z},\dot{\theta },\dot{\varphi },{\dot{Z}}_{M1}\sim {\dot{Z}}_{M6}\right]}^{T}\\ Y_{M}={\left[\ddot{Z},\ddot{\theta },\ddot{\varphi },(Z_{M1}-Z_{m1})\sim (Z_{M6}-Z_{m6})\right]}^{T} \end{array}\right.$$

where the estimated values Z, θ, and φ were obtained by IMU/GPS mounted on the vehicle body; and $Z_{Mi}-Z_{mi}$ were obtained from the direct measurement of active suspension actuator displacement.

Control methods have been proposed to optimize Equation (23) to improve vehicle stability. There are two factors that needed to be considered as the suspension-control method of our vehicle. First, our vehicle, being heavy-duty, has strict actuator-performance limits to ensure driving safety. For another, the estimated terrain map needs to be used more effectively, rather than just as a reference index for path planning. Model predictive control was chosen as the actuator-control method because it not only considers actuator limitations in the process of vehicle optimization, but can also effectively apply terrain information to improve control effect. Several model predictive controllers for vehicle active suspension systems have been proposed [21,28], but they did not give a detailed description of estimating elevation map. In this paper, we proposed the method for road profile estimation and model predictive control based on reference [27]. What is more, we gave outdoor experiment results rather than simulation analysis.

To design a model prediction controller, Equation (22) requires first-order Taylor expansion:

(24) $${\dot{X}}_{M}(r)=f(X_{r},U_{r},W_{r})$$

(25) $${\dot{X}}_{M}(t)=f(X_{r},U_{r},W_{r})+\frac{\partial f}{\partial X}(X-X_{r})+\frac{\partial f}{\partial U}(U-U_{r})++\frac{\partial f}{\partial W}(W-W_{r})$$

Equations (24) and (25) can be rewritten as follows:

(26) $${\dot{\tilde{X}}}_{M}(t)=A_{M}{\tilde{X}}_{M}+B_{M}{\tilde{U}}_{M}+E_{W}{\tilde{W}}_{M}$$

where ${\dot{\tilde{X}}}_{M}(t)={\dot{X}}_{M}(t)-{\dot{X}}_{M}(r)$**,** ${\tilde{X}}_{M}=X-X_{r}$, ${\tilde{U}}_{M}=U-U_{r}$**,** ${\tilde{W}}_{M}=W-W_{r}$

Then, Equation (26) needs to be linearized as follows:

(27) $$\frac{{\tilde{X}}_{M}(k+1)-{\tilde{X}}_{M}(k)}{T}=A_{M}{\tilde{X}}_{M}(k)+B_{M}\tilde{U}(k)+E_{W}\tilde{W}(k)$$

With Equation (27), the following equation can be obtained:

(28) $${\tilde{X}}_{M}(k+1)={\tilde{A}}_{M}{\tilde{X}}_{M}(k)+{\tilde{B}}_{M}{\tilde{U}}_{M}(k)+{\tilde{E}}_{W}{\tilde{W}}_{M}(k)$$

where ${\tilde{A}}_{M}=I+TA_{M}$**,** ${\tilde{B}}_{M}=TB_{M}$**,** ${\tilde{E}}_{W}=TE_{W}$.

According to Equation (28), the prediction of system states can be given by:

(29) $$\begin{array}{l} {\tilde{X}}_{M}(k+1|k)={\tilde{A}}_{M}{\tilde{X}}_{M}(k|k)+{\tilde{B}}_{M}{\tilde{U}}_{M}(k|k)+{\tilde{E}}_{W}{\tilde{W}}_{M}(k|k)\\ {\tilde{X}}_{M}(k+2|k)={\tilde{A}}_{M}{\tilde{X}}_{M}(k+1|k)+{\tilde{B}}_{M}{\tilde{U}}_{M}(k+1|k)+{\tilde{E}}_{W}{\tilde{W}}_{M}(k|k+1)\\ {}^{}{}^{}{}^{}{}^{}{}^{}{}^{}{}^{}{}^{}{}^{}{}^{}{}^{}\vdots \\ {\tilde{X}}_{M}(k+N|k)={\tilde{A}}_{M}{\tilde{X}}_{M}(k+N-1|k)+{\tilde{B}}_{M}{\tilde{U}}_{M}(k+N-1|k)+{\tilde{E}}_{W}{\tilde{W}}_{M}(k|k+N-1) \end{array}$$

With Equations (24) and (29), the following equation can be obtained:

(30) $$\hat{X}=\left[\begin{array}{l} {\tilde{A}}_{M}\\ {\tilde{A}}^{2}{}_{M}\\ \cdots \\ {\tilde{A}}^{N}{}_{M} \end{array}\right]{\tilde{X}}_{M}(k)+\left[\begin{array}{cccc} {\tilde{B}}_{M} & 0 & \cdots & 0\\ {\tilde{A}}_{M}{\tilde{B}}_{M} & {\tilde{B}}_{M} & \cdots & 0\\ \vdots & & & \vdots \\ {\tilde{A}}_{M}{}^{N-1}{\tilde{B}}_{M} & {\tilde{A}}_{M}{}^{N-2}{\tilde{B}}_{M} & \cdots & {\tilde{B}}_{M} \end{array}\right]\hat{U}+{\tilde{E}}_{W}\hat{W}$$

(31) $$\hat{Y}=C_{M}\hat{X}+D_{M}\hat{U}$$

(32) $$\hat{X}=\left[\begin{array}{l} \tilde{X}(k+1)\\ \tilde{X}(k+2)\\ \tilde{X}(k+3)\\ \ldots \\ \tilde{X}(k+N) \end{array}\right],\hat{Y}=\left[\begin{array}{l} \tilde{Y}(k+1)\\ \tilde{Y}(k+2)\\ \tilde{Y}(k+3)\\ \ldots \\ \tilde{Y}(k+N) \end{array}\right],\hat{U}=\left[\begin{array}{l} \tilde{U}(k)\\ \tilde{U}(k+1)\\ \tilde{U}(k+2)\\ \ldots \\ \tilde{U}(k+N-1) \end{array}\right],\hat{W}=\left[\begin{array}{l} \tilde{W}(k)\\ \tilde{W}(k+1)\\ \tilde{W}(k+1)\\ \ldots \\ \tilde{W}(k+N-1) \end{array}\right]$$

A generic model predictive controller finds the optimal sequence of control inputs $\hat{U}$ to minimize cost function $J_{MPC}$:

(33) $$\begin{array}{c} \min^{}J_{MPC}=\min^{}\left({\hat{Y}}^{T}P_{1}\hat{Y}+{\hat{X}}^{T}P_{2}\hat{X}+{\hat{U}}^{T}P_{3}\hat{U}\right)\\ {\hat{U}}_{\min}\leq \hat{U}\leq {\hat{U}}_{\max}\\ \Delta {\hat{U}}_{\min}\leq \Delta \hat{U}\leq \Delta {\hat{U}}_{\max} \end{array}$$

where $P_{1}$, $P_{2}$, and $P_{3}$ are weight matrices of which the parameters can be adjusted to achieve different performance improvements for the vehicle, such as passenger ride comfort, handling stability, and driving safety.

In this study, our objective is to minimize $\ddot{Z}$, $\ddot{\theta }$, and $\ddot{\varphi }$ to maintain the stability of the vehicle. With Equation (32), the suspension control inputs $\hat{U}$ could be calculated and applied to the suspension control.

## 4. Discussion

Three experiments were carried out to demonstrate the effectiveness and feasibility of our method. The first experiment was to confirm that the proposed MPC method improved the stability of the vehicle compared with the output-feedback-control (OFC) method and a passive suspension system. OFC is a common suspension-control method that uses IMU data as a reference index to adjust the active suspension system. The second experiment was to verify that our method for road profile estimation reduces the drift of LiDAR data, thus obtaining a more accurate and stable elevation map. The third experiment was carried out on continuous uneven terrain to verify the stability of our method for a long period.

**Figure 7:**
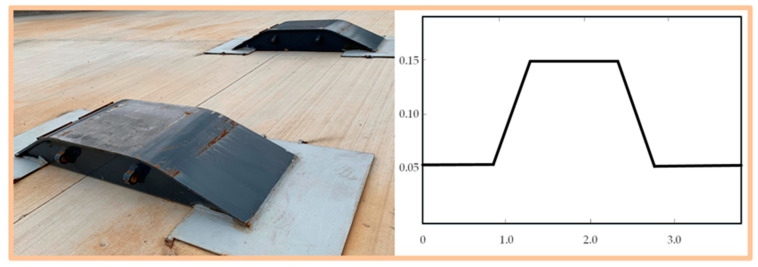
Obstacle at test site.

**Figure 8:**
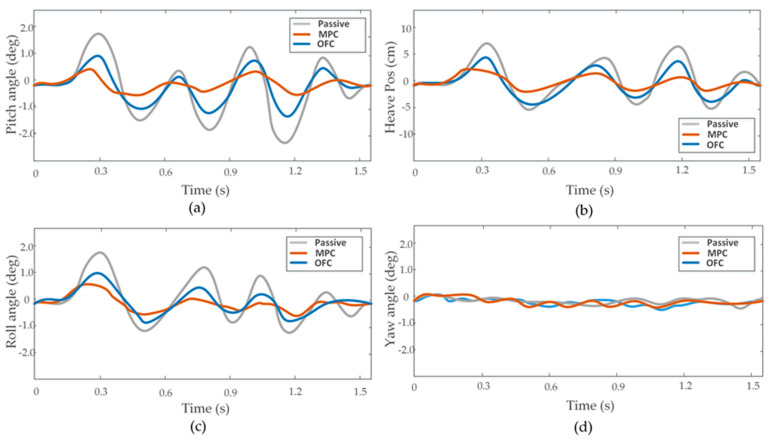
Time domain plots of heave, pitch, roll, and yaw data obtained by sensors. (**a**) Pitch angle. (**b**) Heave position. (**c**) Roll angle. (**d**) Yaw angle.

Experiments were carried out on the test site with some obstacles, as shown in Figure 7. To carry out the first experiment, we made our vehicle directly pass an obstacle using a passive suspension system, MPC method, and OFC method, respectively. The attitude angle and position information obtained by IMU/GPS is shown in Figure 8. Both the MPC and OFC methods reduced the attitude-angle and vertical-displacement values compared with the passive suspension mode. The root-mean-square (RMS) values of displacement and angle for the vertical, pitch, and roll motions are given in Table 1. RMS values of Z, θ, and φ for the OFC method decrease by 37%, 41%, and 46%, whereas reductions for the MPC were 71%, 70%, and 63% when the vehicle was running at a speed of 35 km/h. These results confirmed the efficiency of the proposed MPC method. Other laboratory data with the vehicle running at a speed of 45 km/h are also shown Table 1. As the results demonstrated, the effectiveness of the two control methods was reduced when the speed of the vehicle increased. However, the MPC method still had a good performance in terms of vehicle stability because it calculated the road input in advance on the basis of the estimated elevation map, which ensured that the input value of the actuators could be calculated in advance.

**Figure 9:**
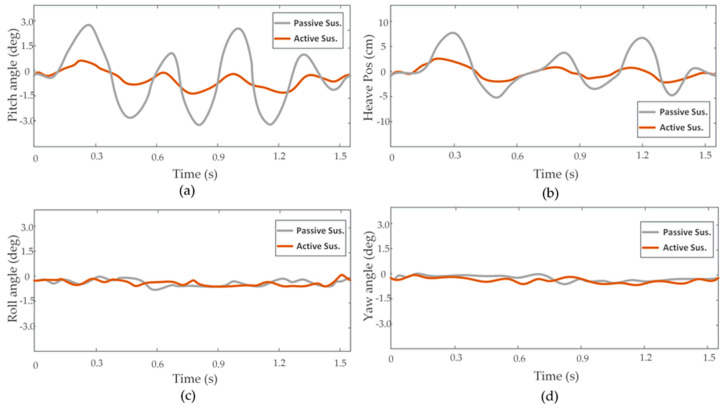
Time domain plots of heave, pitch, roll, and yaw data obtained by sensors. (**a**) Pitch angle. (**b**) Heave position. (**c**) Roll angle. (**d**) Yaw angle.

**Figure 10:**
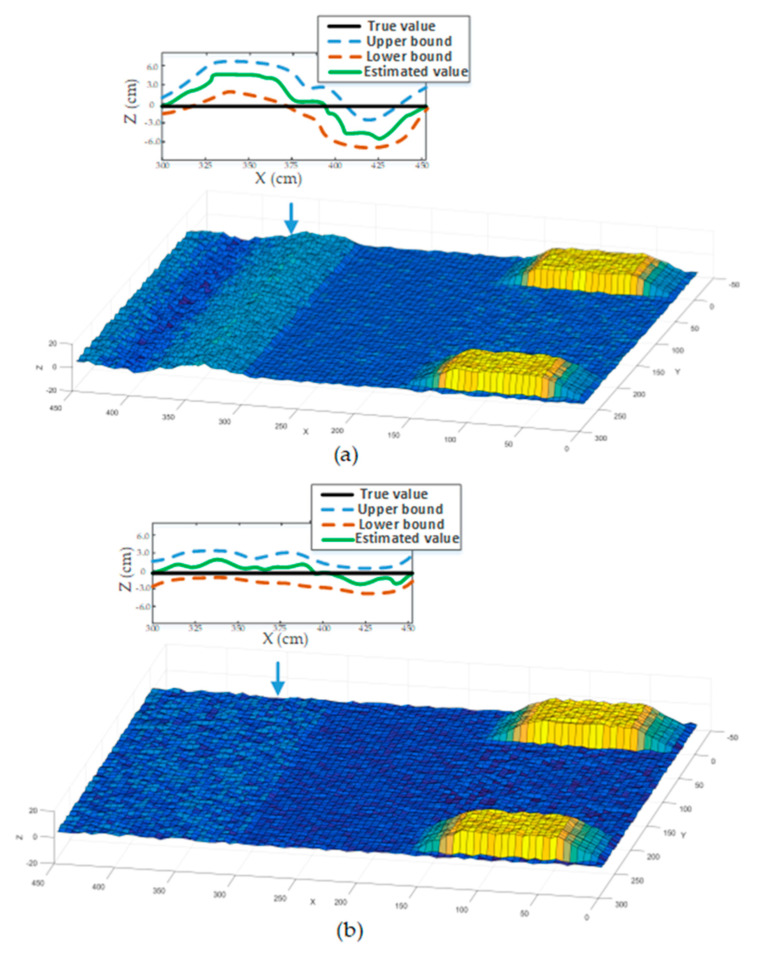
Estimated terrain elevation map. (**a**) Obtained terrain elevation map in passive suspension mode. (**b**) Obtained terrain elevation map in active suspension mode.

In order to verify the accuracy of our method for road profile estimation, we made the vehicle pass two obstacles at the same speed using the active and passive suspension system, respectively. The attitude angle and position information obtained by IMU/GPS is shown in Figure 9. Results demonstrated the improvement of vehicle stability using MPC methods, which is the same as the conclusion of previous experiment results. It is difficult to display details of the elevation terrain since cloud data points obtained by LiDAR are very dense, so we imported LiDAR data into MATLAB by means of interpolation technology to show more details. Figure 10a shows the road profile when the vehicle was passing through obstacles in passive suspension mode. When the front wheels of the vehicle passed the obstacle, the vibration of the vehicle body caused drifts of LiDAR data, which caused a large error in the local elevation map (x = 300–450 cm). Details of the local elevation map (x = 300–450, y = 200 cm) are displayed in the local view of Figure 10a. Blue and red lines represent the upper and lower bounds of the confidence interval, respectively, and the green line represents the estimated elevation value. It has been seen that the maximal error of the estimated elevation value was ~5.68 cm, and the confidence interval was not guaranteed to contain the true elevation values. Figure 10b shows the road profile when the vehicle was passing through obstacles in active suspension mode. Results showed that LiDAR drifts were effectively suppressed due to the improvement of vehicle stability. The local view also shows that the maximal error of the estimated elevation value was ~2.20 cm, and the true elevation values were in the confidence interval. These findings further confirm the efficiency and accuracy of the proposed method for road-profile estimation.

**Figure 11:**
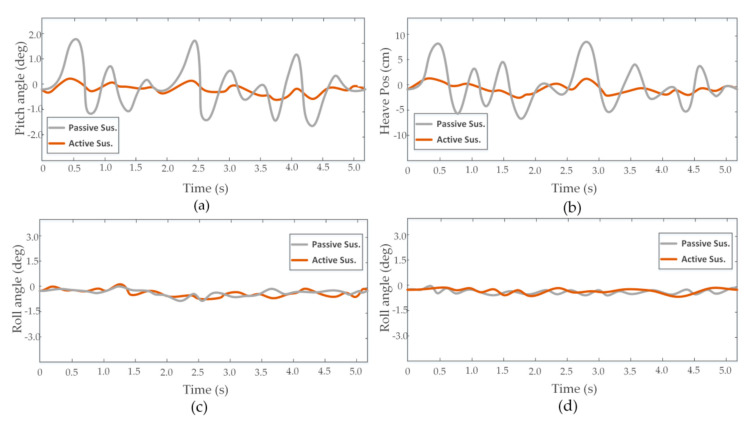
Time domain plots of heave, pitch, roll, and yaw data obtained by sensors. (**a**) Pitch angle. (**b**) Heave position. (**c**) Roll angle. (**d**) Yaw angle.

**Figure 12:**
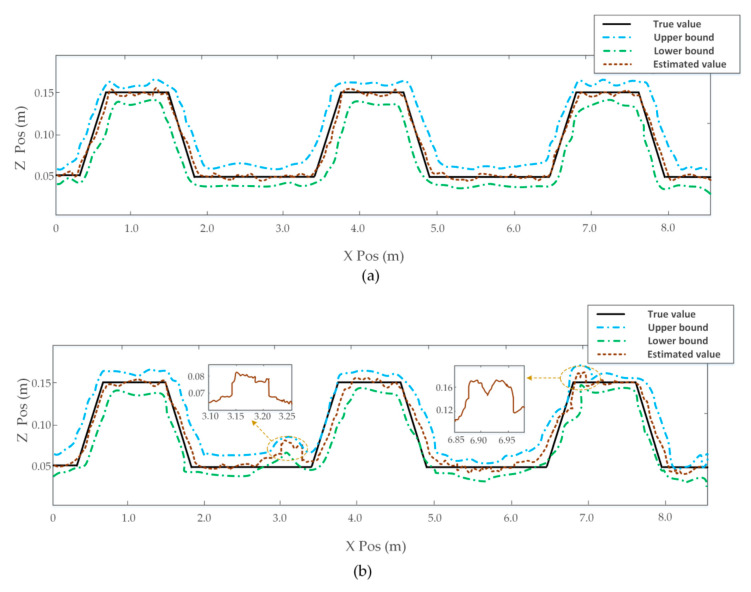
Estimated terrain elevation value. (**a**) Obtained data in active suspension mode. (**b**) Obtained data in passive suspension mode.

To demonstrate the stability of our method for continuous uneven terrain, we made the vehicle pass through a row of obstacles in the active and passive suspension modes, respectively. Attitude angle and position information is shown in Figure 11. Results illustrated that our proposed method still had good stability performance in the suppression of vehicle vibration in the case of continuous road disturbances. Figure 12a shows the local elevation map (x = 0–850, y = 200 cm) when the vehicle was using the active suspension mode. The estimated elevation value approximated the real terrain value, with the maximal error of the estimated elevation value being about 2.15 cm. Figure 12b shows estimated elevation information when the vehicle was using the passive suspension mode. It has been seen that the instability of the vehicle body was aggravated in a continuous-uneven-terrain environment, resulting in more serious drifts of LiDAR data. Furthermore, the Mahalanobis distance criterion also lost its effect in certain positions, which results in cloud data points in the same cell not being able to be effectively fused.

## 5. Conclusions

This paper proposed a novel method for road profile estimation using LiDAR, IMU/GPS, and a vehicle with an active suspension system. The first was the method to obtain the probability elevation terrain using GPS/INS and 3D laser scanners. The second was the description of a model predictive control method, which fused the IMU/GPS and obtained an elevation map to adjust the vehicle suspension system to maintain the stability of the vehicle. Real experiment results demonstrated that the safety and stability of the vehicle driving on complex uneven terrain could be ensured. The elevation information estimation using our method was also more accurate and stable because it avoided the drifts of LiDAR data. There were also some limitations in the whole study. For example, GPS data is unstable under certain conditions, which led to an imprecise location. Further, the model predictive control method needed a higher computational load compared with other control methods, which limited its application in engineering practice. In the future, we will consider how to further improve the accuracy of elevation maps and vehicle stability on complex roads.

## Figures and Tables

**Table 1:** Root-mean-square (RMS) values of vehicle heave, pitch, and roll at different speeds.

Velocity	Type	Passive	OFC	MPC
35 km/h	Heave (cm)	4.13	2.61 (−37%)	1.22 (−71%)
	Pitch (deg)	1.26	0.74 (−41%)	0.39 (−70%)
	Roll (deg)	1.01	0.53 (−46%)	0.37 (−63%)
45 km/h	Heave (cm)	5.05	3.68 (−27%)	1.66 (−67%)
	Pitch (deg)	1.72	0.62 (−30%)	0.61 (−64%)
	Roll (deg)	1.61	0.61 (−31%)	0.64 (−60%)
